# The Effect of Expanded Vermiculite on the Fire Resistance of Waterborne Acrylic Coatings

**DOI:** 10.3390/polym16162302

**Published:** 2024-08-15

**Authors:** Sihong Jiang, Jihu Wang, Shaoguo Wen, Kaimin Chen, Jianlong Zhou, Haopeng Wang, Xuying Deng

**Affiliations:** 1 College of Chemistry and Chemical Engineering, Shanghai University of Engineering Science, Shanghai 201620, China; tony8906@126.com (S.J.); wangjihu@sues.edu.cn (J.W.); kmchen@sues.edu.cn (K.C.); 13293087591@163.com (H.W.); 18249632024@163.com (X.D.); 2 MeToo New Materials Technology (Shanghai) Co., Ltd., Shanghai 201605, China; pz1218@126.com

**Keywords:** fire-retardant coatings, expanded vermiculite, steel structure, smoke suppression

## Abstract

Due to their ability to prevent or slow the spread of fires, fire-retardant coatings are utilized as the main means of fire protection for steel structures, combining easy application and high economic efficiency. This study investigates the effects of the particle size and dosage of expanded vermiculite (EV) on the fire resistance and application performance of coatings. Ammonium polyphosphate, melamine, and pentaerythritol were used as intumescent fire-retardant systems, along with waterborne hydroxyl-modified acrylic resins as the film-forming substances. The properties of fire resistance coatings were tested via scanning electron microscope (SEM), X-ray diffractometry (XRD), thermogravimetric analysis (TGA), limiting oxygen index (LOI), and cone calorimetry. The excellent fire performance of the coatings with 3 wt.% 300-mesh EV was proven, exhibiting a relative expansion of 30.43 times. Moreover, the surface structure of the charcoal layer was dense. The total smoke production (TSP) and smoke concentration (TSR) were only 0.18 m^2^ and 0.25 m^2^/m^2^.

## 1. Introduction

Due to their light weight, high strength, and green and easy construction, steel structures are widely used as the main structural elements in various projects such as supertall buildings, industrial structures, and civil buildings [1,2,3,4]. Although steels are non-combustible materials, the structural strength of common carbon steels rapidly drops at temperatures exceeding 600 °C. Therefore, the fire protection of steel structure buildings has been widely paid attention to. Intumescent fire-retardant coatings are commonly used as fire protection measures for steel buildings. They form a porous and dense charcoal layer after being heated by fire, isolating internal and external oxygen, combustible gases, and heat to protect the steel [5]. A typical intumescent fire protection system includes ammonium polyphosphate (APP) as the acid source, pentaerythritol (PER) as the charcoal-forming agent, and melamine (MEL) as the blowing agent. 

In intumescent fire-retardant coatings, various inorganic functional fillers are used to improve the fire performance of the coating, in addition to reducing the cost of typical coatings. For example, titanium dioxide (TiO_2_) is widely used in intumescent fire-retardant coatings [6]. Rutile titanium dioxide can enhance thermal stability and oxidation resistance [7]. The cross-linking reaction between calcium carbonate (CaCO_3_) and the degradation products of APP can act as a binder to help the formation of the skeleton of the charcoal layer [8]. Additionally, wollastonite with a smaller particle size (CaSiO_3_) can enhance the expansion properties of the char layer when the coating is burned [9]. In addition, several studies have shown that functional fillers can significantly enhance the fire performance of intumescent coatings. The magnesium hydroxide can effectively isolate the transfer of oxygen, heat, and flammable gases between the gaseous and condensed phases during combustion [10]. Zirconium silicate [11], aluminum hydroxide [12], and zinc borate [13] help to enhance the carbon formation and thermal stability of the coatings and have certain smoke suppression properties. Zinc borate and antimony trioxide can also act synergistically to allow the condensed phase to absorb a large amount of heat during the melting process, facilitating the formation of a more stable char residue at elevated temperatures [14,15]. Some carbon structural materials can also enhance the smoke suppression properties of coatings. Glass flakes can limit the migration of APP and PER when exposed to water, enhancing the swelling effect of the coating while improving its water resistance [16]. Expandable graphite forms a porous structure that can absorb a large amount of irritant gases generated by the combustion of the coating. Graphene can promote the formation of a dense and intact carbon layer, thus preventing the diffusion of smoke. This effect may be related to the specific surface area of graphene [17,18]. Carbon black and carbon nanotubes can improve the barrier effect of residual carbon and thus inhibit smoke release [19]. An appropriate amount of clamshell biofillers can provide a good synergistic smoke inhibition effect on fire-retardant coatings, promoting the formation of more residual charcoal and reducing the generation of combustible gases and smoke particles during combustion [20]. High-temperature ceramic fibers and minerals can improve the structural strength of the expanded char layer [21].

Vermiculite is a thermally stable mineral used as a filler and thickener in aqueous coatings to enhance rheology and texture. It has also been found that coatings filled with vermiculite can improve fire resistance and weathering properties, as well as synergize with the intumescent layer to form a more homogeneous, fire-resistant charcoal layer in the event of fire [22].

This study focused on examining the impact of various particle sizes and dosages of EV on the performance of a fire-retardant coating. In this regard, the investigation of EV particle size is easily overlooked, which may affect its arrangement and density in the coating and have an impact on the fire resistance of the coating. Water-based acrylic resin was chosen as the film-forming substance, while APP, PER, and MEL were selected as the fire-retardant intumescent systems. Additionally, titanium dioxide and calcium carbonate were used as fillers. Various tests such as fire resistance tests, SEM, XRD, TGA, and cone calorimetry tests (CCT) were conducted to evaluate the performance of the intumescent fire-retardant coatings and their carbon layers.

## 2. Materials and Methods

### 2.1. Materials

The main materials used in this study are shown in Table 1. 

### 2.2. Preparation of Fire-Retardant Coatings

A mixture of 10 wt.% titanium dioxide, 18 wt.% ammonium polyphosphate, 8 wt.% pentaerythritol, 8 wt.% melamine, 1 wt.% calcium carbonate, and expanded vermiculite (1 wt.%, 2 wt.%, 3 wt.%, 4 wt.%, and 5 wt.%) were dispersed in deionized water, wherein the expanded vermiculite comprised different particle sizes. After dispersing at 1000 rpm for 1 h, 25 wt.% acrylic resin and the appropriate amount of thickener was added and dispersed at 400 rpm for 30 min to produce a water-based vermiculite acrylic fire-retardant coating. The formulations of the coatings are shown in Table 2.

The coating was applied to the polished and cleaned steel plate (150 mm × 80 mm × 0.7 mm) using a coating film preparator. The samples were dried at room temperature for ten days and then tested. The dry film thickness of the coating was controlled at 1.5 ± 0.2 mm.

### 2.3. Characterization

**Figure 1:**
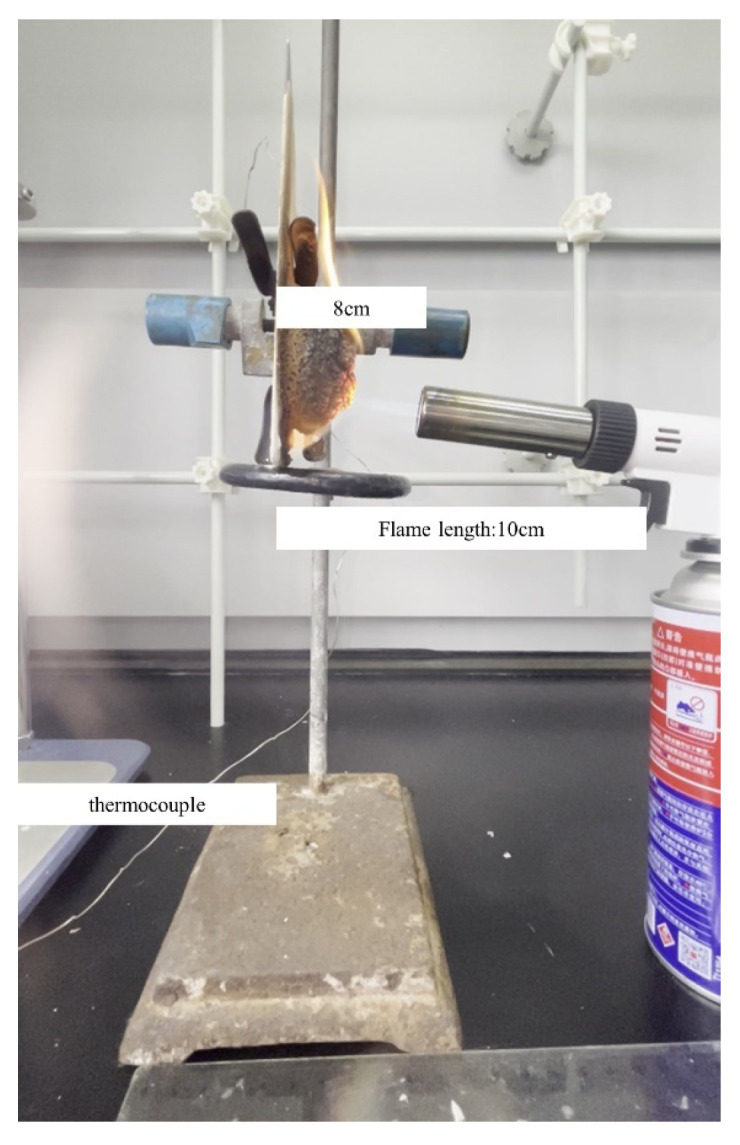
The fire performance test setup.

The fire performance test setup for the coatings is shown in Figure 1. A butane spray gun was used as the flame source, the test samples were placed vertically, and the backboard temperature was monitored using a K-type thermocouple. The distance between the spray gun mouth and the surface of the test samples was 8 cm. A stopwatch was used to record the test times. The thickness of the pre-test coating and post-test charcoal layer were measured and recorded using a steel ruler and noted as l_0_ and l_1_, respectively, and the ratio of l_0_/l_1_ was noted as relative expansion.

The micro-morphology of the charcoal layers was characterized via scanning electron microscopy (SEM, HITACHI SU8010, Tokyo, Japan). The crystal structure of the surface of the charcoal layer was analyzed using X-ray diffractometry (XRD, X Perp PRO, Almelo, The Netherlands) with a Cu K_α_ radiation source (λ = 0.15406 nm). The thermal stability of the coatings was examined using a thermogravimetric analyzer (TGA, TA TGA55, New Castle, DE, USA) with a heating rate of 20 °C/min, an airflow rate of 50 mL/min, and a temperature range of 25 °C to 800 °C. A butane spray gun (516a, Chihiro, Yuhuan, China) was used as the heat source for the fire performance test. A fully automatic oxygen index tester (HAD-LFY-605, Heng Ao, Beijing, China) was used to characterize the limiting oxygen index of the coatings, with a sample size of 14 mm × 5 mm × 0.2 mm. A cone calorimeter (CCT, VOUCH-6810, Jiangsu, China) was used to test the fire retardant and smoke suppression properties of the coatings. 

## 3. Results and Discussion

### 3.1. Fire Protection Properties of the Coatings

#### 3.1.1. Analysis of Time-to-Backboard Temperature Results in Fire Tests

**Figure 2:**
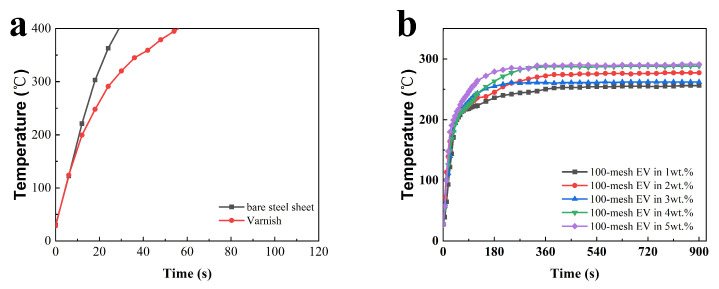

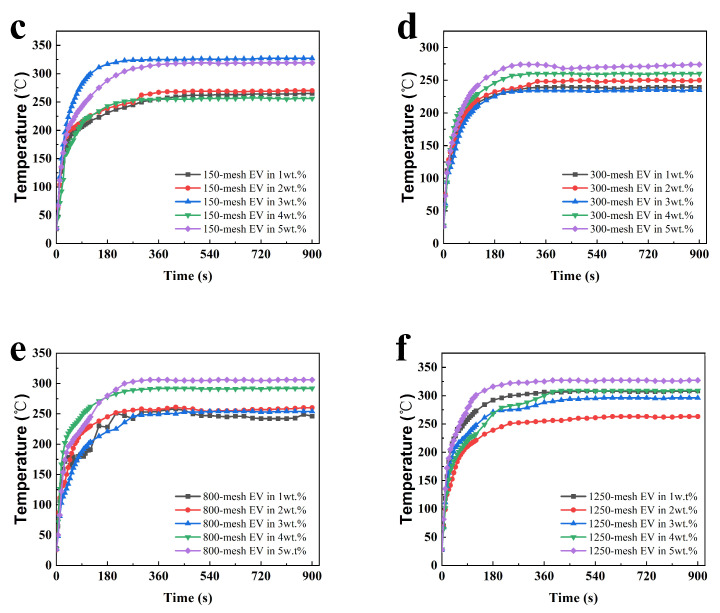
Time-to-backboard temperature profiles of fire performance tests for different intumescent fire-retardant coating films with (**a**) the bare and varnished steel sheets (**b**) 100-mesh EV, (**c**) 150-mesh EV, (**d**) 300-mesh EV, (**e**) 800-mesh EV, and (**f**) 1250-mesh EV.

To analyze the fire protection properties of the coatings more intuitively, the time-to-backboard temperature curves were plotted, as shown in Figure 2. Figure 2a exhibits the fire performance of uncoated steel sheets and steel sheets coated with acrylic varnish, and Figure 2b–f represent the temperature rise of each coating in the fire performance test.

It can be observed in Figure 2a that the temperature of the bare and varnished steel sheets rapidly warmed up to 400 °C in about 30 s and 60 s, respectively, which indicates that EV fire-retardant coatings have a certain fire protection effect on steel plates. After reaching a certain backing plate temperature under a single fire source, the rate of temperature rise is controlled. All samples' time-to-backboard temperature temperatures no longer significantly rise after 360 s. The rate of temperature rise is controlled, and they can protect the steel plate substrate. According to Figure 2d, it can be seen that the T_max_ of the coatings with 300-mesh EV addition is lower overall and the fire performance is better than that of EV with other particle sizes, which indicates that the EV with 300 mesh has better thermal insulation performance in this system.

#### 3.1.2. Analysis of Time-to-Relative Expansion Results in the Fire Tests

**Figure 3:**
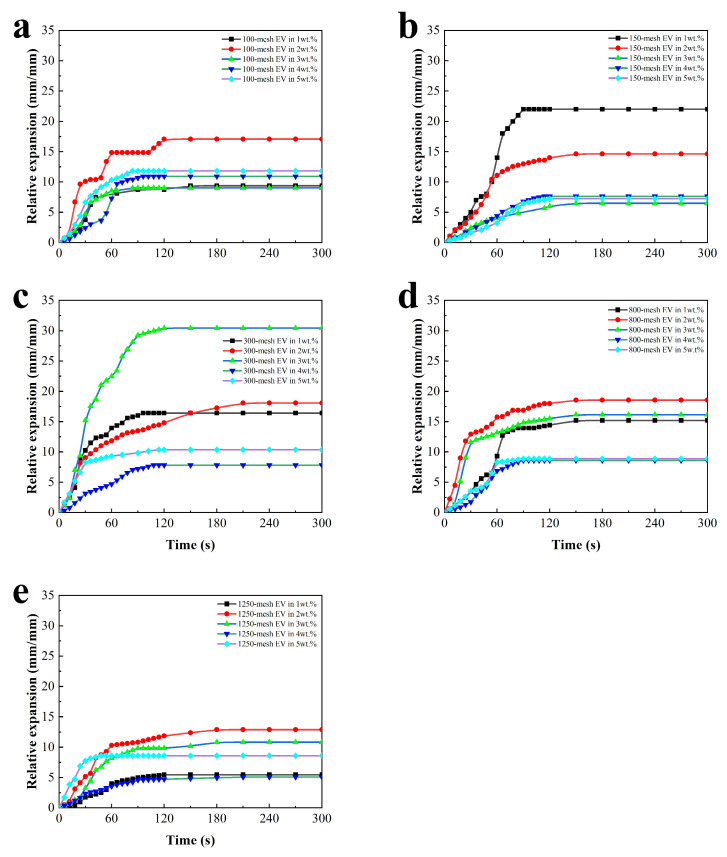
Time-to-relative expansion for intumescent fire-retardant coatings with (**a**) 100-mesh EV, (**b**) 150-mesh EV, (**c**) 300-mesh EV, (**d**) 800-mesh EV, and (**e**) 1250-mesh EV.

To compare and analyze the fire protection properties of the coatings even further, the time-to-relative expansion curves and the side view of the coatings after the fire test are shown in Figure 3 and Table 3, respectively.

It can be seen from Figure 3 that the charcoal layer of the EV fire-retardant coating expanded rapidly during the pre-fire period, swelling to the limit between 60 s and 180 s. The expansion of the charcoal layer was not a linear process. Compared with different sizes of 100-, 150-, 300-, 800-, and 1250-mesh EV, it can be seen that the relative expansion height and expansion rate first increased and then arrived at the highest rate. It can also be seen that too high addition will decrease the relative expansion. The reason for the changing expansion rate may be that the reaction rates of the catalyst, carbon forming agent, and blowing agent within the coating change with the increase in the coating surface temperature until the blowing agent reaction is complete and the carbon layer stops expanding. This indicates that an appropriate EV can help the coating form a porous carbon layer, which can better protect the steel substrate. When the coating is filled with 3 wt.% 300-mesh EV, the relative expansion rate reaches its maximum, reaching 30 mm/mm.

It can be observed in Table 3 that there is a layer of white inorganic material on the surface of the charcoal layer, which may be formed by the gradual oxidation of the organic components within the coating as the coating is continuously heated, which can protect the charcoal layer from burning [7]. Comparing the expansion of the charcoal layer, the coatings with EV additions of 2 wt.% and 3 wt.% showed more significant charcoal layer expansion, which may indicate that the moderate amount of EV helps the coatings to form a porous charcoal layer and provides better protection for the substrate. The expansion of the charcoal layer was lower for the coatings with EV additions of 4 wt.% and 5 wt.%. This may be due to the accumulation of too much EV on the surface of the coating film, which plays a negative role in the expansion of the charcoal layer and significantly reduces the thermal insulation efficiency of the charcoal layer. 

### 3.2. Carbon Layer Surface Morphology

**Figure 4:**
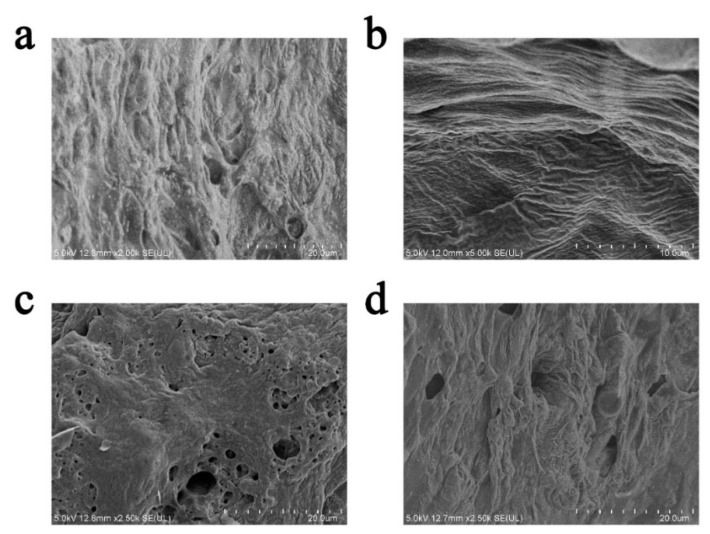
SEM images of an expanded charcoal layer of coatings with (**a**) 300-mesh EV in 1 wt.%, (**b**) 300-mesh EV in 3 wt.%, (**c**) 300-mesh EV in 5 wt.%, and (**d**) 800-mesh EV in 2 wt.%.

The physical structure of the expanded carbon layer has a significant effect on the fire efficiency of the expanded layer [23]. Several typical charcoal layer samples after the fire test were selected, and we observed their morphology via SEM. For these samples, the coatings with 300-mesh EV with better fire protection properties and 800-mesh EV at 2 wt.% with high relative expansion were selected. The results are shown in Figure 4.

From Figure 4, it can be seen that the EV fire-retardant coating expands and undergoes slight shrinkage. Additionally, small cracks and holes are present on the surface of the charcoal layer. These holes may be caused by gas residues formed by the blowing agent when the coating is exposed to fire [24]. As shown in Figure 4a, the char layer of the 300-mesh EV addition of 1 wt.% coating has a tight and compact structure, and this crack-like structure may have a protective effect on the char layer. In Figure 4b,d, the coatings with 300-mesh EV at 3 wt.% and 800-mesh EV at 2 wt.% have a more complete and dense structure. In contrast, in Figure 4c, the carbon layer of the 300-mesh EV at 5 wt.% coating has a loose and porous structure and does not have a dense structure anymore. It is shown that excessive EV in the coating has a negative effect on the structure of the carbon layer.

### 3.3. XRD Analysis of the Carbon Layer Surface

**Figure 5:**
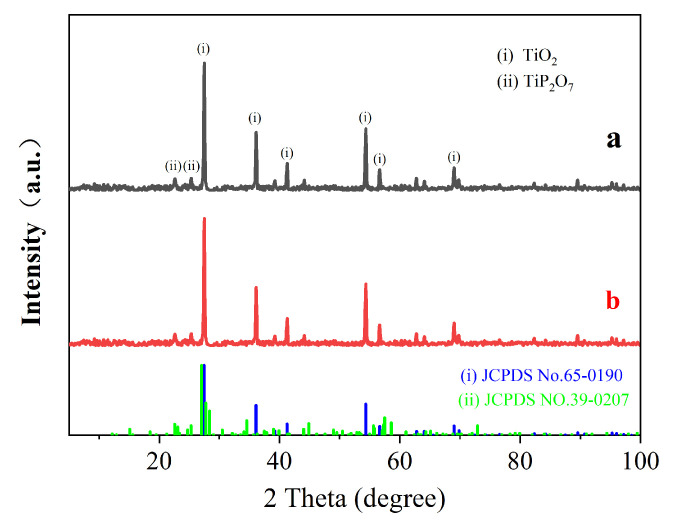
XRD spectra of the expanded carbon layer of coatings (**a**) without EV and (**b**) with 300-mesh 3 wt.% EV.

XRD was used to analyze the structure of the coated expanded carbon layer for the coatings with 300-mesh EV at 3 wt.%, and the results are shown in Figure 5.

Upon analyzing the spectra in Figure 5a,b, the characteristic peaks of the charcoal layer were found to be similar to rutile TiO_2_ and pyrophosphate TiP_2_O_7_ [7]. The peaks at 2 Theta up to 27.5°, 36.3°, and 54.4° are the characteristic peaks of TiO_2_, while the peaks at 22.6° and 25.3° are attributed to TiP_2_O_7_, according to JCPDS NO.65-0190 and JCPDS NO.39-0207. The peaks at 22.6° and 25.3° are attributed to TiP_2_O_7_. The results show that the surface of the carbon layer contains both TiO_2_ and TiP_2_O_7_ as the main phases, confirming the interaction between APP and TiO_2_. The incorporation of EV has a negligible effect on the alteration of the crystalline phase structure on the surface of the carbon layer.

### 3.4. Thermal Analysis of Fire-Retardant Coatings

**Figure 6:**
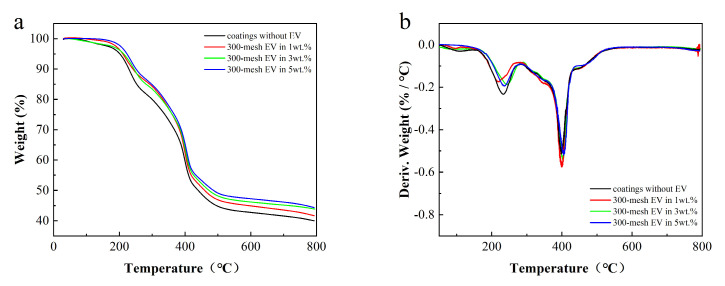
TGA (**a**) and DTG (**b**) curves of fire-retardant coatings.

To analyze the thermal stability of the EV fire-retardant coatings, TGA and DTG curves of coatings without EV and 300-mesh EV contents of 1, 3, and 5 wt.% were constructed, as shown in Figure 6. Table 4 lists the thermal performance parameters of the coatings, where T_5%_ is the temperature at which the coating loses 5% of its weight, T_max_ is the temperature at which the coating reaches the maximum rate of thermal weight loss, and PMLR is the peak of mass loss rate.

As can be seen in Figure 6, the coating mainly experienced three decomposition stages. The first stage is at 30~185 °C, mainly corresponding to the volatilization of water and other VOCs in the coating [25]. The second stage, ranging from 185 °C to 400 °C, is dominated by heat weight loss, accounting for approximately 40%. This corresponds to the thermal degradation of flame retardants and the decomposition of the resin main chain. The third segment occurs between 400 °C and 800 °C, corresponding to the decomposition of CaCO_3_ fillers in the coating [24].

Further compared with Table 4, it can be seen that the addition of EV enhances the stability of the coating. The T_5%_ and T_max_ increased with the increase in EV content, resulting in slower decomposition throughout the process. Adding EV increases the amount of residual carbon in the coating, enhancing its thermal stability and char formation. A higher amount of residual carbon also improves the smoke suppression performance of the coatings.

### 3.5. Limiting Oxygen Index of Fire-Retardant Coatings

**Figure 7:**
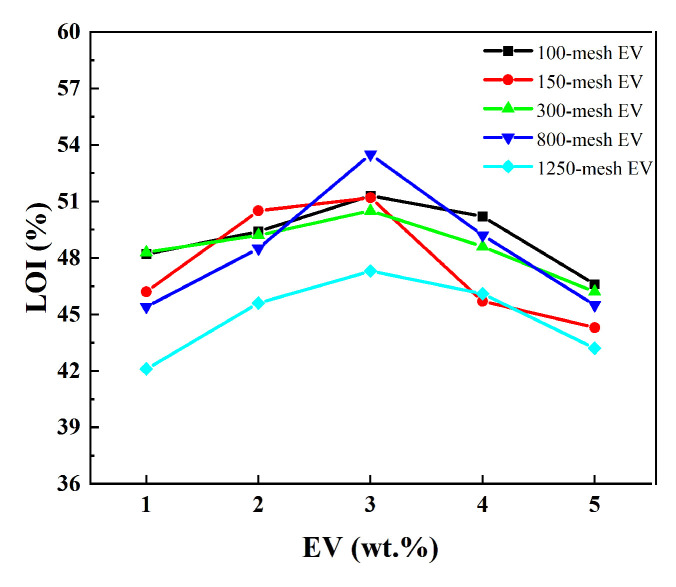
LOI of different intumescent fire-retardant coatings with EV.

The test results on the limiting oxygen index of the fire-retardant coatings are presented in Figure 7.

Figure 7 indicates that the limiting oxygen index of the EV fire-retardant coating exhibited a trend of initially increasing and then decreasing with the rise in EV content. The highest limiting oxygen index of the coating, at 53.5%, was observed when 3 wt.% of 800-mesh EV was added. When the addition was 3 wt.% of 100-, 150-, and 300-mesh EV, the coatings had the same LOI. But it was only lower by about 1.5%. Since the fire prevention mechanism of intumescent fire-retardant coatings involves the synergistic coordination of its components, it achieves the expansion and heat insulation of the charcoal layer. Therefore, the appropriate amount of EV can effectively synergize with the acrylic base material, charcoal-forming agent, and foaming agent to create a high-quality heat-insulating layer. Excessive addition of EV causes aggregation and may lead to insufficient charring and foaming of the coating, as well as inhibiting its flame-retardant properties. Considering the cost, and the relationship between content and performance, 3 wt.% 300-mesh EV was chosen for other analyses. 

### 3.6. Cone Calorimetry Tests for Fire-Retardant Coatings

**Figure 8:**
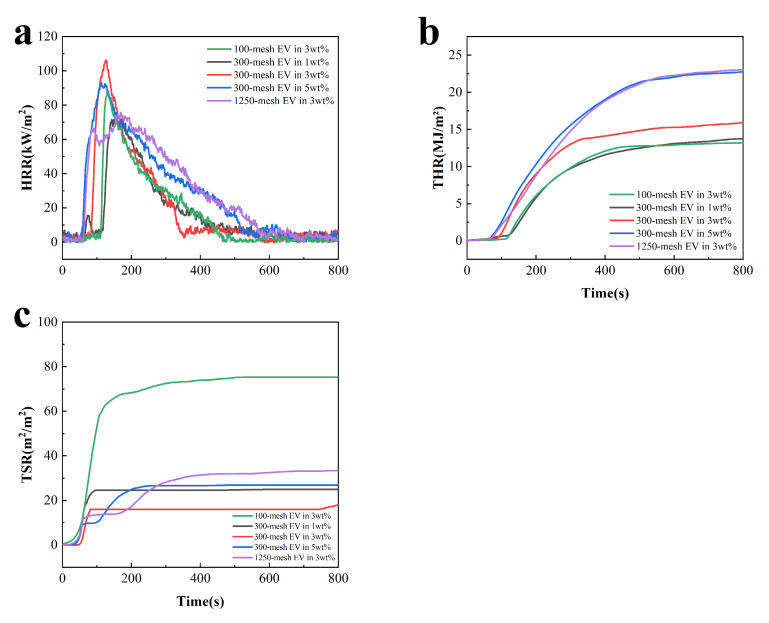
CCT curves of fire-retardant coatings containing different charring agents at a heat flux of 35 kW/m^2^: (**a**) HRR, (**b**) THR, and (**c**) TSP.

Cone calorimetry testing (CCT) evaluates the fire retardant and smoke suppression properties of fire-resistant coatings. It provides a wealth of combustion data for coatings, including time to ignition (TTI), heat release rate (HRR), peak heat release rate (P-HRR), total heat release rate (THR), total smoke production (TSP), and smoke concentration (TSR). Figure 8 and Table 5 display the cone calorimetric data for coatings with different particle sizes and additions of EV. Samples with different additions of 300-mesh EV were used to test cone calorimetry. For comparison, 100- and 1250-mesh EV samples with 3 wt.% additions were selected.

From Figure 8, at the initial stage of the test, as the coating was gradually pyrolyzed to ignition, the HRR increased rapidly and the first exothermic peak appeared at about 70 s. The temperature continued to rise, and the coating began to foam and expand to form an expanded char layer. At this stage, the expanded charcoal layer is important in isolating oxygen and slowing heat transfer. When the surface of the charcoal layer continues to oxidize and rupture at high temperatures, a large amount of gas escapes from inside the coating, and the second peak of HRR occurs at about 120 s. With the selected EV at 300 mesh, the coating exhibited lower TSP and TSR values of 0.18 m^2^ and 0.25 m^2^/m^2^, respectively. This indicated that the char layer formed by the selected 300-mesh EV with 3 wt.% addition could play a better role in flame retardancy, heat insulation, and smoke suppression.

According to the data in Table 5, the coatings with 300-mesh EV addition of 5 wt.% and 1250-mesh EV addition of 3 wt.% have higher THR and TSR, which may be attributed to the increased distribution of EV on the surface of the coatings. The charcoal layer has more pores and cracks at high temperatures, allowing for efficient heat transfer and smoke to the outside. In comparison with other coatings, the TTI of the coating with 3 wt.% EV addition was longer, consistent with the test results in Figure 7, further indicating that with 3 wt.% EV addition, the EV has good synergy with the acrylic base and flame-retardant system, and the coating has excellent flame-retardant properties. Subsequently, the charcoal layer foamed and expanded to form a heat-insulating layer, resulting in a higher peak exothermic value. Following the formation of the insulating char layer, gas diffusion inside the coating could be effectively prevented.

## 4. Conclusions

This study examined the potential use of expanded vermiculite (EV) in waterborne steel structure fire-retardant acrylic coatings. In particular, it analyzed the properties of different particle sizes and amounts of EV on the fire-retardant coatings. The performed fire tests revealed that EV addition is conducive to enhancing the fire performance of the coating. At an EV mesh size and dosage of 300-mesh and 3 wt.%, respectively, the coating had better fire performance (the backing temperature of 247 °C and the highest intumescent multiplication ratio of 30.43 times). The TGA proved that the EV enhanced the thermal stability and the charcoal-forming properties of the coating. The oxygen index proved that the coating had the best flame-retardant property at the EV dosage of 3 wt.%. The cone calorimetry test verified that the coating had the best smoke-suppression property at the EV mesh size and dosage of 300-mesh and 3 wt.%, respectively, corresponding to TSP = 0.18 m^2^ and TSR = 0.25 m^2^/m^2^. These findings are considered instrumental in fire prevention via flame-retarding coatings.

## Figures and Tables

**Table 1:** The main raw materials list.

Materials	Type	Producers
Water-based hydroxyl-modified acrylic resin	Industrial grade	Qixiang Qingchen New Material Technology Co., Ltd., Shanghai, China.
Expandable vermiculite (100, 150, 300, 800, and 1250 mesh)	Industrial grade	Qingdao Qianshuo International Trade Co., Ltd., Qingdao, China.
Calcium carbonate	Industrial grade	Hebei Hongyao Mineral Products Processing Factory, Shijiazhuang, China.
Titanium dioxide	Industrial grade	Yangzhou Dilan Chemical Materials Co., Ltd., Yangzhou, China.
Ammonium polyphosphate	Chemically pure	Fengxi Fertiliser Group Co., Ltd., Yuncheng, China.
Pentaerythritol	Chemically pure	Sinopharm Group Chemical Reagent Co., Ltd., Shanghai, China.
Melamine	Industrial grade	Shanghai Xumiao Non-halogenated Smoke Reduction and Flame Retardant Co., Ltd., Shanghai, China.
Thickener	Industrial grade	Haimens Deqian (Shanghai) Chemical Co., Ltd., Shanghai, China.

**Table 2:** Formula of vermiculite acrylic fire-retardant coating.

Materials	Mass Fraction (wt.%)
Water-based hydroxyl-modified acrylic resin	25.0
Expandable vermiculite (100, 150, 300, 800, and 1250 mesh)	1.0; 2.0; 3.0; 4.0; 5.0
Calcium carbonate	1.0
Titanium dioxide	10.0
Ammonium polyphosphate	18.0
Pentaerythritol	8.0
Melamine	8.0
Thickener	Appropriate amount
Deionized water	Appropriate amount

**Table 3:** Side view of different coated carbon layers for different intumescent fire-retardant coating films with 100-, 150-, 300-, 800-, and 1250-mesh EV.

	Dosage of EV				
Mesh	1 wt.%	2 wt.%	3 wt.%	4 wt.%	5 wt.%
100	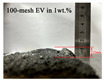	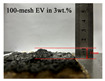	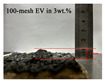	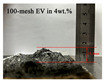	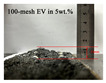
150	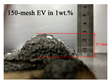	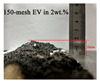	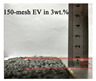	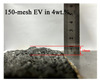	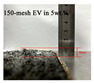
300	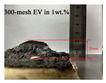	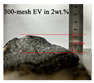	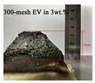	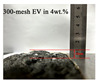	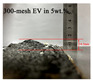
800	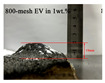	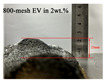	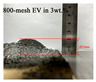	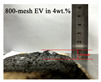	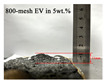
1500	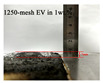	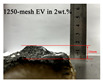	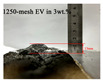	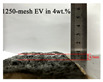	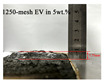

**Table 4:** Thermal parameters of intumescent fire-retardant coatings.

M_0_ (Mesh)	W (wt.%)	T_5%_ (°C)	T_max_ (°C)	PMLR (%/min)	Residue Weight (%)
/	0	201	398	0.515	40.0
300	1	210	399	0.576	41.7
	3	213	404	0.587	43.9
	5	223	405	0.521	44.3

**Table 5:** CCT data of intumescent fire-retardant coatings.

M_0_ (Mesh)	W (wt.%)	TTI (s)	P-HRR (kW/m^2^)	THR (MJ/m^2^)	TSP (m^2^)	TSR (m^2^/m^2^)
100	3	107	88.52	13.36	0.77	17.62
300	1	54	74.44	14.06	0.22	18.25
300	3	81	106.55	16.51	0.18	0.25
300	5	57	93.27	23.28	0.24	18.76
1250	3	59	76.29	23.39	0.30	22.82

## Data Availability

The original contributions presented in the study are included in the article, further inquiries can be directed to the corresponding author.
